# Birth weight, cardiometabolic risk factors and effect modification of physical activity in children and adolescents: pooled data from 12 international studies

**DOI:** 10.1038/s41366-020-0612-9

**Published:** 2020-06-03

**Authors:** Guro Pauck Bernhardsen, Trine Stensrud, Bjørge Herman Hansen, Jostein Steene-Johannesen, Elin Kolle, Wenche Nystad, Sigmund Alfred Anderssen, Pedro C. Hallal, Kathleen F. Janz, Susi Kriemler, Lars Bo Andersen, Kate Northstone, Geir Kåre Resaland, Luis B. Sardinha, Esther M. F. van Sluijs, Mathias Ried-Larsen, Ulf Ekelund, on behalf of the International Children’s Accelerometry Database (ICAD) Collaborators

**Affiliations:** 1 Department of Sports Medicine, Norwegian School of Sport Sciences, Oslo, Norway; 2 Department of Sport Science and Physical Education, University of Agder, Kristiansand, Norway; 3 Chronic Diseases and Aging, Norwegian Institute of Public Health, Oslo, Norway; 4 Federal University of Pelotas, Pelotas, Brazil; 5 Department of Health and Human Physiology, University of Iowa, Iowa City, Iowa USA; 6 Epidemiology, Biostatistics and Public Health Institute, University of Zürich, Zürich, Switzerland; 7 Department of Sport, Food and Natural Sciences, Campus Sogndal, Western Norway University of Applied Sciences, Sogndal, Norway; 8 Population Health Sciences, Bristol Medical School, University of Bristol, Bristol, UK; 9 Center for Physically Active Learning, Faculty of Education, Arts and Sports, Campus Sogndal, Western Norway University of Applied Sciences, Sogndal, Norway; 10 Exercise and Health Laboratory, CIPER, Faculty of Human Kinetics, Universidade de Lisboa, Lisbon, Portugal; 11 Centre for Diet and Activity Research (CEDAR) & MRC Epidemiology Unit, University of Cambridge, Cambridge, UK; 12 Centre for Physical Activity Research, Rigshospitalet Copenhagen, Capital Region of Denmark, Copenhagen, Denmark

**Keywords:** Paediatrics, Epidemiology, Risk factors

## Abstract

**Objectives:**

Low and high birth weight is associated with higher levels of cardiometabolic risk factors and adiposity in children and adolescents, and increases the risk of cardiovascular diseases, obesity, and early mortality later in life. Moderate-to-vigorous physical activity (MVPA) is associated with lower cardiometabolic risk factors and may mitigate the detrimental consequences of high or low birth weight. Thus, we examined whether MVPA modified the associations between birth weight and cardiometabolic risk factors in children and adolescents.

**Methods:**

We used pooled individual data from 12 cohort- or cross-sectional studies including 9,100 children and adolescents. Birth weight was measured at birth or maternally reported retrospectively. Device-measured physical activity (PA) and cardiometabolic risk factors were measured in childhood or adolescence. We tested for associations between birth weight, MVPA, and cardiometabolic risk factors using multilevel linear regression, including study as a random factor. We tested for interaction between birth weight and MVPA by introducing the interaction term in the models (birth weight x MVPA).

**Results:**

Most of the associations between birth weight (kg) and cardiometabolic risk factors were not modified by MVPA (min/day), except between birth weight and waist circumference (cm) in children (*p* = 0.005) and HDL-cholesterol (mmol/l) in adolescents (*p* = 0.040). Sensitivity analyses suggested that some of the associations were modified by VPA, i.e., the associations between birth weight and diastolic blood pressure (mmHg) in children (*p* = 0.009) and LDL- cholesterol (mmol/l) (*p* = 0.009) and triglycerides (mmol/l) in adolescents (*p* = 0.028).

**Conclusion:**

MVPA appears not to consistently modify the associations between low birth weight and cardiometabolic risk. In contrast, MVPA may mitigate the association between higher birth weight and higher waist circumference in children. MVPA is consistently associated with a lower cardiometabolic risk across the birth weight spectrum. Optimal prenatal growth and subsequent PA are both important in relation to cardiometabolic health in children and adolescents.

## Introduction

The developmental origin of health and disease concept (DOHaD) suggests that fetal and infant life could be critical periods for the development of cardiovascular diseases and obesity later in life [1]. A large number of studies have provided support for the impact of a low birth weight, used as a proxy measure of fetal growth restriction, on subsequent risk of cardiovascular diseases in adulthood [2, 3]. Moreover, a high birth weight is consistently associated with increased risk of obesity later in life [4].

Low physical activity (PA) is another important and widely recognized risk factor for cardiovascular diseases [5]. A recent harmonized meta-analysis showed a nonlinear risk reduction in all-cause mortality across PA at any intensity in middle-aged and older people [6]. The greatest risk reduction was observed at the lower end of the PA continuum; hence, the proposed public health message is “sit less, move more and more often” [6].

Cardiovascular diseases develop gradually and they rarely manifest in childhood or adolescence; nevertheless, even at young ages early signs of disease are apparent and birth weight is inversely associated with cardiometabolic risk factors [7–9] and positively associated with risk of obesity [10]. Furthermore, PA–especially at higher intensities [11–13]–is consistently associated with lower cardiometabolic risk factors in the general population of children and adolescents. However, it is unknown if PA modifies the association between birth weight and cardiometabolic risk factors [14], with two previous studies in children and adolescents including only a few cardiometabolic outcomes showing contradictory results [15, 16].

We hypothesized that higher moderate-to-vigorous PA (MVPA) may mitigate the associations between birth weight and cardiometabolic risk factors in children and adolescents. Examining children and adolescents may be of particular interest since interventions early in life may provide an opportunity for early intervention well before cardiovascular diseases manifest. Furthermore, device-measured PA, more specifically accelerometers, are considered the method of choice when examining associations between intensities of PA and health outcomes in children and adolescents [17, 18]. The aim of this study was, therefore, to examine whether device-measured MVPA modifies the associations between birth weight and several cardiometabolic risk factors in a diverse sample of children and adolescents. By testing the statistical interaction between birth weight and MVPA on these associations we also effectively examined whether MVPA is associated with cardiometabolic health across the birth weight spectrum.

## Materials and methods

### Study design and participants

We used pooled individual data from nine studies included in the International Children’s Accelerometry Database (ICAD) [19], a subcohort of the Norwegian Mother, Father and Child Cohort Study (MoBa) [20], Physical Activity among Norwegian Children Study (PANCS) [21, 22] and Active Smarter Kids (ASK) [23]. Table 1 lists the number of participants and simple descriptive statistics of participants from each study included in the pooled analyses.

**Table 1:** Study characteristics and descriptive characteristics of participants stratified by study.

Study	Country, city/area	Year	*n* (% boys)	Age, years	BMI, kg/m^2^	>Compulsory education, (%)^a^
ALSPAC	UK, Avon	2006–2008	2110 (45.1%)	15.4 (15.3–15.6)	21.4 (3.6)	89.6
Denmark EYHS	Denmark,Odense	1997–2010	1438 (44.0%)	10.2 (9.6—15.5)	18.9 (3.3)	88.7
Estonia EYHS	Estonia, Tartu	1998–1999	568 (44.2%)	10.2 (9.5–15.4)	18.4 (3.0)	81.2
IBDS	US, Iowa	2003–2005	431 (48.0%)	11.0 (10.9–11.3)	20.0 (4.4)	86.3
Norway EYHS	Norway, Oslo	1999–2000	236 (50.8%)	9.7 (9.4–10.0)	16.9 (2.2)	58.5
Pelotas	Brazil, Pelotas	2006–2007	426 (52.8%)	13.3 (13.1–13.6)	20.3 (3.8)	23.5
Portugal EYHS	Portugal,Madeira	1999–2000	590 (51.5%)	10.0 (9.6–15.4)	19.4 (3.7)	6.5
SPEEDY	UK, Norfolk	2011	358 (45.5%)	14.3 (14.0–14.5)	20.8 (3.9)	68.1
KISS	Switzerland^b^	2005–2006	306 (45.8%)	10.4 (7.1–11.2)	17.3 (2.8)	92.7
MoBa	Norway^c^	2013–2015	430 (54.4%)	11.0 (10.3–11.3)	17.8 (2.4)	96.9
ASK	Norway^d^	2014	857 (51.6%)	10.2 (10.0–10.5)	18.1 (3.1)	97.9
PANCS	Norway^e^	2005–2006	1350 (49.9%)	9.8 (9.5–15.3)	18.6 (3.3)	94.8

Data are expressed as mean (*SD*) for BMI, median (25th–75th percentile) for age, number of participants (*n*), percent boys (%) and percent >compulsory education.

*ALSPAC* Avon Longitudinal Study of Parents and Children, *ASK* Active Smarter Kids, *BMI* body mass index, *EYHS* European Youth Heart Study, *IBDS* The Iowa Bone Development Study, *KISS* The Kinder-Sportstudie, *MoBa* the Norwegian Mother, Father and Child Cohort Study, *PANCS* Physical Activity Among Norwegian Children Study, *SPEEDY* Sport, Physical Activity and Eating Behavior: Environmental Determinants in Young People Study.

^a^Percent (%) of which one or both parents have completed any post-compulsory education.

^b^Two provinces in Switzerland.

^c^Four cities in Norway (Oslo, Fredrikstad, Stavanger, and Bergen).

^d^Sogn and Fjordane county.

^e^Representative sample from all regions in Norway.

Results from three of the studies included in ICAD on the associations between birth weight and insulin and waist circumference, and effect modification of MVPA, have previously been published [16]. In the present study, we extend the study with a more than fourfold increase in the number of participants for insulin and more than sevenfold for waist circumference, and by including additional cardiometabolic variables.

ICAD [19] consists of device-measured PA, anthropometrics, and health data collected in children and adolescents from 20 studies worldwide. A detailed description of the aims, design, recruitment of studies, and protocols of the ICAD project have been described in detail elsewhere [19], and the harmonization documents are available at the ICAD website (http://www.mrc-epid.cam.ac.uk/research/studies/icad/data-harmonisation/). For the present analyses, we used data from nine ICAD-studies (ICAD 2.0). Three studies are prospective birth-cohort studies [24–27] and six are cross-sectional studies with retrospectively reported birth weight [28–30]. In longitudinal studies, data from the first wave of which each person participated is included, unless later waves of data collection comprised a wider array of cardiometabolic risk factors [24, 26, 27, 29]. The participants were recruited either from being born at a certain hospital or area in a specific period [24–27], through randomly selected schools [28] or through schools willing to participate within a defined area [29, 30]. More information about the population and recruitment method in each study is available elsewhere [24–30].

MoBa is an ongoing prospective population-based pregnancy cohort study conducted by the Norwegian Institute of Public Health [20]. Participants were recruited from all over Norway from 1999 to 2008. The women consented to participation in 40.6% of the pregnancies. We invited a subcohort of 1603 10-12-year-old from four areas in Norway, of which 430 children participated and provided sufficient data for the present analyses.

The PANCS 1 study included a nationally representative sample of Norwegian 9- and 15- year-old [21, 22]. In total 2299 agreed to participate, and the participation rate was 89 and 74% for 9- and 15- year-old, respectively.

The ASK study is a school-based cluster randomized controlled trial carried out in 2014/15, situated in the western part of Norway [23]. Sixty schools were approached and 57 schools (1129 children) agreed to participate (recruitment success of 95% of schools, 94% of children). For the present analyses, we included the baseline data on PA and cardiometabolic risk factors in 857 children.

Each collaborator in the ICAD consulted their research board to make sure sufficient ethical approval had been obtained. Written informed consent was obtained from each child’s parent prior to all testing in ICAD, the subcohort of MoBa, PANCS, and ASK, and the study protocols were approved by the Regional Committee for Medical Research Ethics.

### Measurements

#### Birth weight

Birth weight was either measured at birth [20, 24–27] or retrospective parentally reported [21–23, 28–30].

### Cardiometabolic risk factors

Eleven studies provided data on systolic- and diastolic blood pressure (Table 2) [20–23, 25–30]. Blood pressure was measured repeatedly in a resting condition using automated blood pressure monitors, and the mean of repeated measures (two or three) was calculated. Eight studies (Table 2) provided data on LDL-cholesterol, HDL-cholesterol, and triglycerides [21–23, 26–28, 30]. In one study, [28] fasting blood samples were drawn from capillary blood. Fasting glucose and insulin levels were available from seven studies (Table 2) [21–23, 26–28, 30]. We calculated insulin resistance (Homeostatic model assessment, HOMA-IR) using the updated HOMA2 calculator [31]. All blood samples were collected while participants were in a fasting state. All twelve studies included data on waist circumference [20–30]. Test-personnel measured waist circumference midway between the lower rib and the iliac crest [20–22, 24–30], or two cm above the level of the umbilicus [23] at the end of a gentle expiration.

**Table 2:** Descriptive characteristics (mean and *SD* unless otherwise stated^b^) of study participants and study availability, stratified by age group.

	Children		Adolescents	
	Studies^a^	Mean (SD)	Studies^a^	Mean (SD)
No. (n(%boys))^b^	2–5, 7, 9–12	4560 (49.9%)	1–4, 6–10, 12	4540 (45.6%)
Age (years)^b^	2–5, 7, 9–12	9.9 (9.5–10.4)	1–4, 6–10, 12	15.4 (15.1–15.6)
BMI (kg/m^2^)	2–5, 7, 9–12	17.8 (3.0)	1–4, 6–10, 12	21.0 (3.5)*
>compulsory education,%^b,c^	2–5, 7, 9–12	84.1%	1–4, 6–10, 12	76.2%*
Birth weight (kg)	2–5, 7, 9–12	3.51 (0.60)	1–4, 6–10, 12	3.39 (0.57)*
MVPA (min/day)	2–5, 7, 9–12	62.0 (31.8)	1–4, 6–10, 12	44.7 (26.5)*
VPA (min/day)^b^	2–5, 7, 9–12	14.4 (16.7)	1–4, 6–10, 12	10.5 (15.8)*
SBP (mmHg)	2–3, 5, 7, 9–12	102.8 (8.7)	1–3, 6–10, 12	116.5 (12.6)*
DBP (mmHg)	2–3, 5, 7, 9–12	62.3 (8.2)	1–3, 6–10, 12	66.4 (8.9)*
LDL-cholesterol (mmol/l)	2–3, 5, 7, 9, 11–12	2.48 (0.66)	1–3, 7, 9, 12	2.17 (0.60)*
HDL- cholesterol (mmol/l)	2–3, 5, 7, 9, 11–12	1.61 (0.36)	1–3, 7, 9, 12	1.35 (0.31)*
Triglycerides (mmol/l)^b^	2–3, 5, 7, 9, 11–12	0.64 (0.49–0.85)	1–3, 7, 9, 12	0.74 (0.58–0.97)*
HOMA-IR (score)^b^	2–3, 7, 9, 11–12	0.7 (0.5–1.0)	1–3, 7, 9, 12	1.1 (0.8–1.5)*
Waist circumference (cm)	2–5, 7, 9–12	62.5 (8.7)	1–4, 6–10, 12	73.2 (8.9)*

*DBP* diastolic blood pressure, *HDL* high density lipoprotein, *HOMA-IR* Homeostasis Model Assessment (HOMA2), *LDL* low density lipoprotein, *MVPA* moderate to vigorous physical activity, *SBP* systolic blood pressure, *VPA* vigorous physical activity.

*p < 0.05 for difference between children and adolescents.

^a^Studies: 1-ALSPAC, 2-Denmark EYHS, 3-Estionia EYHS, 4-IBDS, 5-Norway EYHS, 6-Pelotas, 7-Portugal EYHS, 8-SPEEDY, 9-KISS, 10-MoBa, 11-ASK, 12-PANCS.

^b^Age, VPA, triglycerides and HOMA-IR expressed as median (25th–75th percentile). Number of participants (%), and % >compulsory education.

^c^Percent (%) of which one or both parents have completed any post-compulsory education.

Clustered risk scores with different combinations of cardiometabolic risk factors are comparable [32], and we therefore used the available variables and calculated a clustered cardiometabolic risk score by summarizing age-group specific standardized values of mean arterial blood pressure (MAP, systolic blood pressure + (diastolic blood pressure*2)/3), triglycerides, LDL/HDL-ratio and HOMA-IR, divided by 4 (number of variables).

### Physical activity

PA was measured at the same time-point as the cardiometabolic risk factors using uniaxial Actigraph- (model GT1M and 7164) [19, 21, 22] and triaxial Actigraph- (model GT3X+) [20, 23] accelerometers. The Actigraph accelerometers are previously validated in free-living conditions among children and adolescents, and are significantly and moderately correlated with physical activity energy expenditure derived from double-labeled water [33, 34]. The monitor was attached around the waist (right hip) using an elastic band. The children and adolescents were told to wear the monitor for four to seven consecutive days, removing it only when sleeping or during water-based activities. For data harmonization purposes, all data were reintegrated into uniaxial format and 60 s epoch. All studies provided raw Actigraph data files and the data were further reanalyzed in a standardized way to ensure comparability across studies (using Kinesoft version 3.3.20 and version 3.3.80). Nonwear time was defined as ≥60 min of consecutive zeros, with an allowance of 2 min of nonzero interruptions. A valid day was defined as at least 480 min of measured wear time, and all children providing at least 3 valid days were included in the analyses. We used two outcome measures of PA–MVPA for main analyses and vigorous PA (VPA) for sensitivity analyses. MVPA was defined as average minutes per day ≥2296 counts per minute (cpm), whereas VPA was defined as average minutes per day ≥4012 cpm [35]. We removed files flagged as spurious in the ICAD-project [19]. The overnight activity was removed [19, 21–23], or days with more than 18 h wear time were set to missing [20].

### Covariates and descriptive variables

Potential confounders included in the models were parental education and sex. We further adjusted for waist circumference (model 2) and height (for systolic- and diastolic blood), measured at follow-up, to examine the direct associations. Ages at follow-up were included in the model to improve the precision of the outcomes due to the known changes in cardiometabolic risk factors with increasing age in these age-groups [32].

Standardized methods were used to measure height and weight across all studies [20–30]. For descriptive purposes, we calculated body mass index (BMI) as weight (in kilograms) divided by height (in meters) squared.

To harmonize the parent’s education level, a dichotomous variable was created dividing the maternal and paternal education level into (1): up to and completion of compulsory education and (2): any post-compulsory education. We further combined the parents’ education variables into one variable reflecting the highest education level by either the mother or the father.

### Statistical analysis

Only participants for whom data for birth weight, PA (≥3 valid days) and at least one cardiometabolic risk factor were available were included in the analyses (=3534 participants removed). The participant’s age distribution revealed two clusters around age 9–10 and 15–16 years. To ensure homogenous groups we therefore performed a median split dividing the participants into children (≤11.6 years old) and adolescents (>11.6 years old) for all analyses. We tested for differences between the age-groups using independent sample *t* test, Mann–Whitney two-sample test, and chi-squared statistics. We used multilevel linear regression, including study as a random factor (12 studies), to examine the associations between birth weight, MVPA, and the cardiometabolic outcomes. We adjusted all models for the highest parental education, age, and sex. When systolic- and diastolic blood pressure were modeled as the outcome we adjusted for childhood height and excluded age from the model due to the risk of collinearity. In model 2, we further adjusted analyses for waist circumference. Furthermore, to examine whether MVPA modified the associations between birth weight and the cardiometabolic outcomes we included the interaction term (birth weight x MVPA) in the models. A significant interaction indicates an additive interaction given the linearity of the model. We tested all models for the assumptions of linear regression (linearity between independent and dependent variables, normal distribution of residuals, and homoscedasticity). For the models including HOMA-IR and triglycerides, a slightly skewed distribution of the residuals was shown. However, due to the large sample size and sensitivity analyses with and without log-transformed variables showing similar results, we kept the variables not transformed in the models to ease the interpretation of the effect estimates. Furthermore, we used robust standard errors estimates due to signs of heteroscedasticity in some of the models. A formal interaction test showed no evidence of an interaction with sex on any of the associations. We conducted sensitivity analyses using VPA as an effect modifier, and sensitivity analyses where we excluded all participants with birth weight <1.5 kg, i.e., participants most likely to be born prematurely (*n* = 66).

In case of a significant interaction (*p* < 0.10) we graphically illustrated the predicted values of the outcome variable, based on the final adjusted models with the interaction term, across values of birth weight and the 25th, 50th, and 75th percentile of MVPA/VPA. Regardless of an interaction, we also graphically illustrated the predicted values of the clustered cardiometabolic risk score in a similar manner.

Three % (*n* = 116) and 19 % (*n* = 875) of the children and adolescents, respectively, had missing data on one or more of the included covariates. We replaced missing values using multiple imputation (MI) with Fully Conditional Specification. We imputed 20 datasets. Further details on participants with missing values, the MI-method, and results from complete case analyses are provided in Supplementary Information (File S1).

We performed all analyses using Stata/SE version 14.1. The two-sided statistical level was set to *p* < 0.05 for associations and *p* < 0.10 for interaction effects.

## Results

Descriptive characteristics of the study sample are provided in Table 2. The participants wore the accelerometer on average for 4.9 (SD = 1.3) and 5.3 (SD = 1.4) days, with an average of 792 (SD = 69.0) and 814 (SD = 89.7) minutes per day, for children and adolescents, respectively.

Higher MVPA was associated with lower systolic- and diastolic blood pressure, LDL-cholesterol, triglycerides, HOMA-IR, waist circumference, and clustered cardiometabolic risk score–except for systolic blood pressure in children and waist circumference in adolescents (Table 3). Higher MVPA was further associated with higher HDL-cholesterol (Table 3).

**Table 3:** Associations (unstandardized regression coefficients and 95%CIs) between both birth weight and MVPA on cardiometabolic risk factors in children and adolescents, and interaction between birth weight and MVPA on the same cardiometabolic outcomes.

	Children			Adolescents		
	*n*	*B*(95%*CI*)	*p* value	*n*	B (95%CI)	*p* value
SBP (mmHg)	4120			4491		
Model 1^a^						
Birth weight (kg)		−1.10 (−1.50, −0.70)			−1.78 (−2.52, −1.04)	
Model 2^b^						
MVPA (min/day)		−0.01 (−0.03, 0.00)			−0.02 (−0.03, −0.01)	
Birth weight (kg)		−1.30 (−1.67, −0.94)			−1.98 (−2.66, −1.30)	
Birth weight x MVPA		0.005 (−0.007, 0.017)	0.440		−0.005 (−0.032, 0.021)	0.685
DBP (mmHg)	4119			4491		
Model 1^a^						
Birth weight (kg)		−0.66 (−0.80, −0.42)			−0.32 (−0.61, −0.04)	
Model 2^b^						
MVPA (min/day)		−0.01 (−0.03, −0.00)			−0.01 (−0.02, −0.00)	
Birth weight (kg)		−0.74 (−0.99, −0.48)			−0.36 (−0.65, −0.08)	
Birth weight x MVPA		−0.004 (−0.011, 0.002)	0.168		−0.002 (−0.023, 0.021)	0.924
LDL–cholesterol (mmol/l)	3225			2868		
Model 1^a^						
Birth weight (kg)		0.03 (−0.00, 0.06)			−0.000 (−0.04, 0.04)	
Model 2^b^						
MVPA (min/day)		−0.001 (−0.002, −0.001)			−0.001 (−0.002, −0.000)	
Birth weight (kg)		0.01 (−0.01, 0.03)			−0.01 (−0.05, 0.03)	
Birth weight x MVPA		0.000 (−0.001, 0.001)	0.915		−0.000 (−0.001, 0.000)	0.202
HDL–cholesterol (mmol/l)	3230			2868		
Model 1^a^						
Birth weight (kg)		−0.02 (−0.05, 0.01)			−0.02 (−0.03, 0.00)	
Model 2^b^						
MVPA (min/day)		0.001 (0.000, 0.002)			0.001 (0.000, 0.001)	
Birth weight (kg)		0.001 (−0.028, 0.030)			−0.004 (−0.019, 0.012)	
Birth weight x MVPA		−0.000 (−0.001, 0.001)	0.978		−0.001 (−0.001, −0.000)	0.040
Triglycerides (mmol/l)	3207			2866		
Model 1^a^						
Birth weight (kg)		−0.01 (−0.03, 0.01)			0.003 (−0.007, 0.012)	
Model 2^b^						
MVPA (min/day)		−0.001 (−0.002, −0.000)			−0.001 (−0.002, −0.000)	
Birth weight (kg)		−0.03 (−0.05, −0.02)			−0.01 (−0.02, −0.00)	
Birth weight x MVPA		−0.000 (−0.001, 0.001)	0.921		0.000 (−0.000, 0.000)	0.839
HOMA-IR (score)	3109			2859		
Model 1^a^						
Birth weight (kg)		−0.01 (−0.05, 0.03)			0.01 (−0.03, 0.05)	
Model 2^b^						
MVPA (min/day)		−0.002 (−0.003, −0.001)			−0.002 (−0.003, −0.001)	
Birth weight (kg)		−0.07 (−0.11, −0.03)			−0.02 (−0.06, 0.00)	
Birth weight x MVPA		0.000 (−0.000, 0.001)	0.689		−0.000 (−0.001, 0.001)	0.941
Waist Circumference (cm)	4536			4129		
Model 1^a^						
MVPA (min/day)		−0.03 (−0.05, −0.02)			−0.01 (−0.03, 0.00)	
Birth weight (kg)		1.90 (1.57, 2.23)			1.55 (0.96, 2.15)	
Birth weight x MVPA		−0.010 (−0.018, −0.003)	0.005		−0.001 (−0.017, 0.015)	0.896
Clustered risk score	3079			2839		
Model 1^a^						
Birth weight (kg)		−0.008 (−0.06, 0.04)			−0.003 (−0.04, 0.03)	
Model 2^b^						
MVPA (min/day)		−0.003 (−0.005, −0.002)			−0.003 (−0.003, −0.002)	
Birth weight (kg)		−0.08 (−0.12, −0.04)			−0.05 (−0.07, −0.02)	
Birth weight x MVPA		−0.000 (−0.001, 0.001)	0.774		0.000 (−0.001, 0.001)	0.948

Separate models for MVPA and birth weight (model 2). When interaction term (birth weight x MVPA) is examined, both MVPA and birth weight are also included in the model.

*DBP d*iastolic blood pressure, *HDL h*igh-density lipoprotein, *HOMA-IR* homeostasis model assessment (HOMA2), *LDL* low-density lipoprotein, *MVPA m*oderate to vigorous physical activity, *SBP s*ystolic blood pressure.

^a^Model 1: Adjusted for highest parental education, sex and age. SBP and DBP adjusted for height instead of age.

^b^Model 2: Adjusted for model 1 and waist circumference.

^c^Clustered cardiometabolic risk score calculated from summing standardized values for MAP (mean arterial blood pressure), triglycerides, LDL/HDL-ratio and HOMA-IR, divided by 4 (number of variables).

**Fig. 1:**
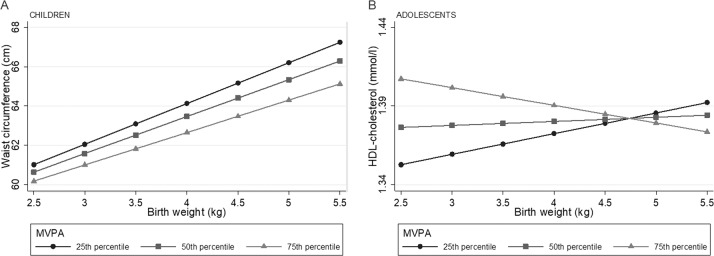
Illustration of the interaction between birth weight and MVPA on waist circumference (children) and HDL-cholesterol (adolescents). Predicted values of cardiometabolic outcomes (**a**: waist circumference, **b**: HDL-cholesterol) across birth weight and the 25th, 50th and 75th percentile of MVPA in children (**a**) and adolescents (**b**) from regression models with significant interaction between birth weight and MVPA (*p* < 0.1). Adjusted for highest parental education, sex, age and waist circumference (when not the outcome). HDL high density lipoprotein, MVPA moderate to vigorous physical activity MVPA children: 25th percentile = 39.6 min/day, 50th percentile = 57.7 min/day, 75th percentile=80.0 min/day. MVPA adolescents: 25th percentile = 25.3 min/day, 50th percentile = 39.8 min/day, 75th percentile = 58.7 min/day.

Lower birth weight was associated with higher systolic- and diastolic blood pressure, an association that became stronger in magnitude after the inclusion of waist circumference in the model (Table 3). Birth weight was not associated with LDL- or HDL-cholesterol, whereas lower birth weight was associated with higher triglyceride levels, HOMA-IR (children only) and clustered cardiometabolic risk score following adjustments for waist circumference (Table 3). Higher birth weight was associated with higher waist circumference. Introducing the interaction term (birth weight x MVPA) into the model suggested an effect modification by MVPA on the association between birth weight and waist circumference in children, and HDL-cholesterol in adolescents (Table 3). Predicted waist circumference increased by higher birth weight in the 25th, 50th, and 75th percentile of MVPA, however, the increase is slightly steeper in the 25th percentile compared with the 75^th^ percentile of MVPA (Fig. 1a). Figure 1b suggests that at the 75th percentile of MVPA the association between birth weight and HDL-cholesterol was negative, whereas the association was positive at the 25th percentile of MVPA.

**Fig. 2:**
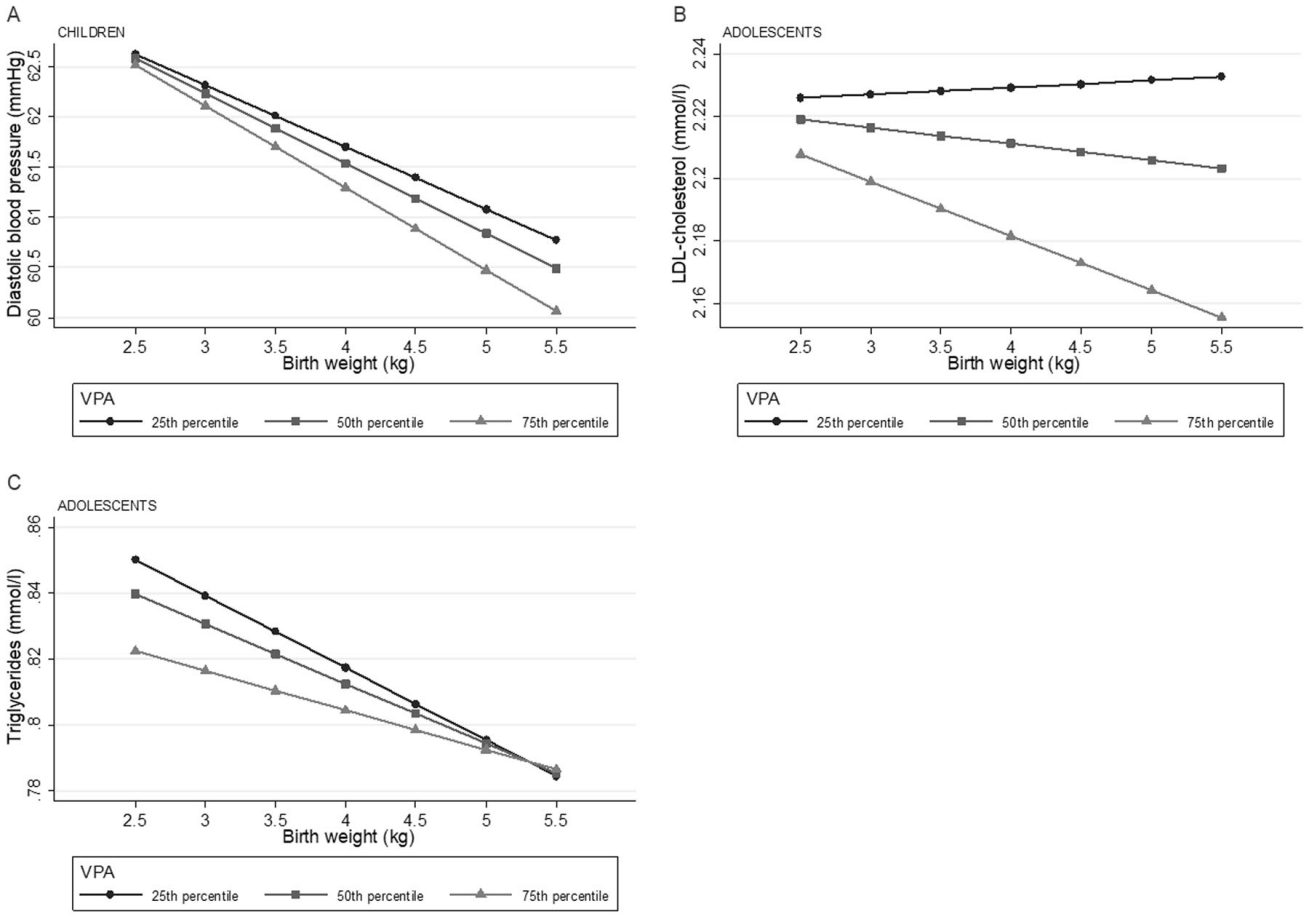
Illustration of the interaction between birth weight and VPA on diastolic blood pressure (children), LDL-cholesterol (adolescents) and triglycerides (adolescents). Predicted values of cardiometabolic outcomes (**a**: diastolic blood pressure, **b:** LDL-cholesterol, **c:** triglycerides) across birth weight and the 25th, 50th, and 75th percentile of VPA in children (**a**) and adolescents (**b, c**) from regression models with significant interaction between birth weight and VPA (*p* < 0.1). Adjusted for highest parental education, sex, age (height for diastolic blood pressure) and waist circumference (when not the outcome). HDL high density lipoprotein, LDL low density lipoprotein, MVPA moderate to vigorous physical activity, VPA vigorous physical activity VPA children: 25th percentile = 7.7 min/day, 50th percentile = 14.4 min/day, 75th percentile = 24.5 min/day. VPA adolescents: 25th percentile = 4.5 min/day, 50th percentile = 10.5 min/day, 75th percentile = 20.3 min/day.

Sensitivity analyses suggested that VPA modified the association between birth weight and diastolic blood pressure in children and between birth weight and LDL-cholesterol and triglycerides in adolescents. These associations are illustrated across the 25th, 50th, and 75th percentile of VPA in Fig. 2. Although lower diastolic blood pressure at the 75th percentile compared with the 25th percentile of VPA, the association between birth weight and diastolic blood pressure was somewhat stronger at the 75th percentile (Fig. 2a). Figure 2b shows that the association between birth weight and LDL-cholesterol in adolescents appeared to be negative at the 75th percentile, and slightly positive at the 25th percentile of VPA. A somewhat steeper negative association was observed at the 25th percentile of VPA compared with the 75th percentile on the association between birth weight and triglycerides in adolescents (Fig. 2c).

**Fig. 3:**
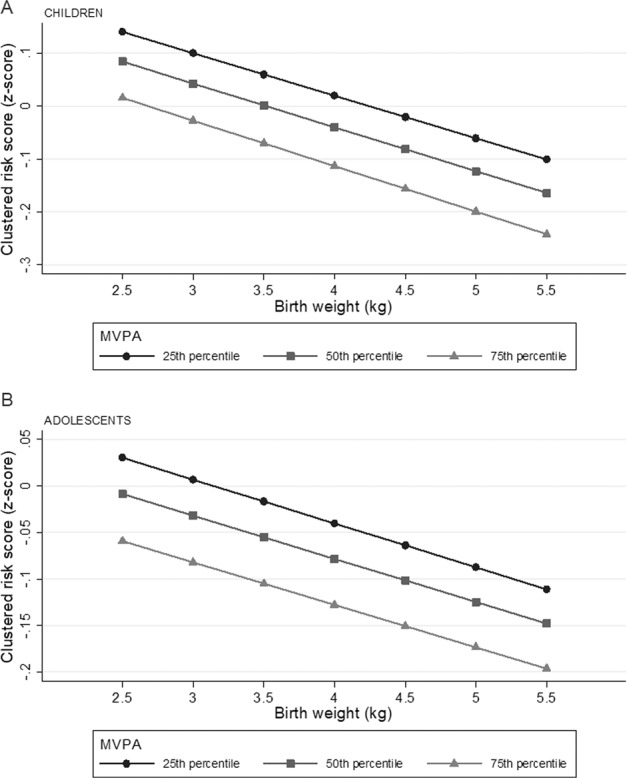
Illustration of the association between birth weight and the clustered cardiometabolic risk score across MVPA (no interaction between birth weight and MVPA). Predicted clustered cardiometabolic risk score across birth weight and 25th, 50th, and 75th percentile of MVPA from regression model with interaction term (birth weight x MVPA) in children (**a**) and adolescents (**b**), *p* value for interaction between birth weight and MVPA; children *p* = 0.774, adolescents *p* = 0.948. Adjusted for highest parental education, waist circumference, sex and age. Clustered cardiometabolic risk score calculated from summing standardized values for MAP (mean arterial blood pressure), triglycerides, LDL/HDL-ratio and HOMA-IR, divided by four (number of variables). MVPA moderate to vigorous physical activity Children MVPA: 25th percentile = 39.6 min/day, 50th percentile = 57.7 min/day, 75th percentile = 80.0 min/day. Adolescents: 25th percentile = 25.3 min/day, 50th percentile = 39.8 min/day, 75th percentile = 58.7 min/day.

Figure 3 illustrates the inverse association between birth weight and the clustered cardiometabolic risk score. The magnitude of the associations was similar across levels of MVPA.

Results from sensitivity analyses excluding participants with birth weight <1.5 kg did not differ from the results including the full birth weight spectrum (data not shown).

The complete case analyses (Supplement File 1) did not differ from the results using MI on missing values, except for a nonsignificant interaction of MVPA and birth weight on the association with HDL-cholesterol in adolescents.

## Discussion

We observed that MVPA does not modify the association between a lower birth weight and an adverse cardiometabolic clustered risk in children and adolescents, nor consistently modify the associations with single risk factors. MVPA may slightly attenuate the association between higher birth weight and higher waist circumference in children. The observed effect modification of MVPA or VPA on diastolic blood pressure, LDL- cholesterol, HDL- cholesterol, and triglycerides are likely clinically insignificant.

Few previous studies have examined a possible effect modification of PA on the association between low birth weight and measures of insulin resistance or risk of type 2 diabetes, with contradictory results. Findings by Ridgway et al. [16], are similar to ours suggesting that device-measured PA appears not to modify the association between birth weight and HOMA-IR in children and adolescents. In a similar study by Ortega et al. [15], a significant interaction was observed between birth weight and device-measured PA for the association between birth weight and HOMA-IR, suggesting the association was attenuated in the most active adolescents. Laaksonen et al. [36] and Eriksson et al. [37] observed that higher self-reported PA attenuated the odds of type 2 diabetes and metabolic syndrome in the low birth weight group in middle-aged men and elderly people. They observed an interaction on the multiplicative (relative risk) scale which may differ from interaction on the additive (risk difference) scale [38]. In contrast, Jeanne et al. [39] observed no interaction on the multiplicative scale between birth weight and self-reported MVPA on risk of diabetes or prediabetes in young adulthood. Our results extend previous observations by including a substantially larger and more heterogeneous sample likely led to more precise effect estimates.

We observed that MVPA may modify the association between birth weight and HDL-cholesterol in adolescents, whereas the highest intensity is necessary to modify the association with LDL-cholesterol and triglycerides. It appears that a lower birth weight is associated with lower LDL-cholesterol, but also lower HDL-cholesterol in the least active, whereas it is in the opposite direction for the more active adolescents. This is in contrast to one study suggesting that an 8-week exercise program may eliminate the difference in LDL-cholesterol between low birth weight and high birth weight in young adulthood, whereas the difference in HDL-cholesterol between the two groups became significantly different first after the exercise period [40]. The observed interactions need to be confirmed in future research to investigate whether they are biased by confounding factors (e.g., pubertal status or nutrition), if birth weight influence the response of PA on lipid levels in adolescents and whether these interactions persist into adulthood and may become more clinically important in the development of cardiovascular diseases.

The observed stronger association between birth weight and diastolic blood pressure in the most active may indicate that children with low birth weight do not respond to VPA to the same extent as children with higher birth weight. This interaction was however not observed in adolescents, nor for systolic blood pressure.

The results from a few previous studies are similar to ours and suggest that self-reported PA attenuates the association between a high birth weight and abdominal adiposity [41] and risk of overweight or obesity [41, 42] in children and adolescents. We observed this interaction in children only, whereas another study observed this interaction in girls only [42]. In contrast, other studies found no interaction between birth weight and PA on the association with abdominal adiposity [16, 42, 43], or other measures of adiposity [43, 44].

A clustered cardiometabolic risk score is likely more important for future health than single risk factors [32]. In contrast to our results, Jeanne et al. [39] observed an interaction between self-reported MVPA and high birth, but not low birth weight, on the association with a cardiovascular disease risk score in young adulthood. Although we did not observe any effect modification of PA, the clustered cardiometabolic risk score is substantially lower in more active compared with less active across the birth weight spectrum, and MVPA should thus be considered an important public health strategy in children and adolescents.

We consider device-measured PA in a large study sample as an important strength of this study, but this method is also prone to misclassification. Children’s PA pattern is sporadic which makes precise measurements difficult [45]; thus, the use of a 60-s epoch length may have led to underestimation of time spent in MVPA [46]. Also, accelerometers underestimates activities with little vertical acceleration of the hip, e.g., bicycling, and water activities due to removal of the monitor. Birth weight is used as a proxy for intrauterine growth and, in some of the included studies, measured using retrospective parental reports. However retrospective parent-reported birth weight show strongly agreement with measured birth weight [47, 48]. Optimally we would have adjusted for gestational age in our analyses. Unfortunately, this information was not available. The results from sensitivity analyses where we excluded participants with birth weight <1.5 kg did not differ from the results using the full study sample; however, this cannot fully compensate for the lack of data on gestational age. PA and the cardiometabolic risk factors are measured at the same time point, which limits our ability to infer causality. This is of particular concern when waist circumference is modeled as the outcome, as it is likely that a higher waist circumference may lead to reduced PA [49, 50].

The main strengths of this study are the large and diverse sample of children and adolescents with available data on several cardiometabolic risk factors and device-measured PA analyzed in a harmonized manner.

## Conclusion

We did not observe strong evidence for a modifying effect of MVPA on the association between birth weight and cardiometabolic risk factors in children and adolescents, although it may to some degree attenuate the association between high birth weight and waist circumference in children. Higher levels of MVPA is consistently associated with a more favorable cardiometabolic risk profile across the birth weight spectrum.

## Supplementary information

Supplemental material File 1
