# Supplementary material for: Birth weight, cardiometabolic risk factors and effect modification of physical activity in children and adolescents: pooled data from 12 international studies
Source: Int J Obes (Lond). 2020 Jun 3;44(10):2052–63. doi: 10.1038/s41366-020-0612-9 (PMC7508671; doi:10.1038/s41366-020-0612-9)
Supplement: Supplementary file 1 — Supplemental material File 1 [file 41366_2020_612_MOESM1_ESM.pdf]

FileS1: Information on imputation method, number of missing values, participants with complete and incomplete data and complete case analyses.

Amount of missing differed between the studies (% with missing on one or more of the covariates= SPEEDY=5.87%, ALSPAC=37.06%, EYHS-Denmark=5.01%, EYHS Estonia= 0.70%, EYHS-Norway=3.81%, EYHS-Portugal=1.86%, KISS=15.03%, Pelotas=0.23%, IBDS=0.70%, ASK=3.03%, PANCS=0.52%, MoBa=2.09%). The large amount of missing in studies including adolescents (ALSPAC) probably explain much of the large difference in amount of missing between children and adolescents. There are some differences between participants with missing and participants with complete data (S1 File-Table 1) on the outcomes.

We used Fully Conditional Specification (FCS), with logistic regression when imputing values on missing on parental education and predictive mean matching (pmm) when imputing values on missing on waist circumference and height (as well as for the outcome variables). All variables in the final models (including the interaction term) were included in the imputation model, in addition to study and country. We conducted the imputation model separately for children and adolescents. We removed participants with missing on the outcome of interest for each analysis model.

We assume that data are missing at random (MAR), given the observed variables that are included in the imputation model.

Table S1: Descriptive characteristics (mean and sd unless otherwise stated) of study participants, stratified by age group and participants with complete and incomplete data.

	CHILDREN		ADOLESCENTS	
	Complete data (n=4533)	Incomplete data (n=117)	Complete data (n=3769)	Incomplete data (n=879)
	Mean (sd)	Mean (sd)	Mean (sd)	Mean (sd)
Birth weight (kg)	3.51 (0.60)	3.43 (0.59)	3.40 (0.57)	3.37 (0.58)
MVPA (min/day)	62.0 (31.8)	63.5 (32.5)	44.6 (26.8)	45.1 (25.1)
SBP (mmHg)	102.8(8.7)	102.8 (9.3)	115.3(12.5)	121.5 (11.7)*
DBP (mmHg)	62.3 (8.2)	61.7 (7.7)	66.2 (9.0)	67.3 (8.5)*
LDL-cholesterol (mmol/l)	2.5 (0.6)	2.4(0.7)	2.2 (0.6)	2.1 (0.6)*
HDL- cholesterol (mmol/l)	1.6 (0.4)	1.6 (0.3)	1.4(0.3)	1.3 (0.3)*
Triglycerides (mmol/l)	0.64(0.36)	0.65(0.40)	0.74(0.40)	0.74(0.38)
HOMA-IR (score)	0.7 (0.5)	0.7 (0.5)	1.1 (0.7)	1.2 (0.7)*
Waist circumference (cm)	62.6 (8.7)	59.1 (8.2)*	72.9(8.9)	75.7(8.9)*

[b]Triglycerides and insulin expressed as median (25-75 percentile)

DBP- Diastolic blood pressure; HDL- High density lipoprotein; HOMA-IR- Homeostasis Assessment Model (HOMA2); LDL- Low density lipoprotein; MVPA –Moderate to vigorous physical activity; SBP- Systolic blood pressure

*p<0.05 for differences between participants with complete- and incomplete data

Table S2: Covariates with missing values and descriptive statistics of complete variables (complete) and the variables with imputed on missing values (MI).

Variable	n missing (%)	Complete	MI
Children			
Parental education	87 (2%)	>compulsory education[a]= 84.1%	>compulsory education[a]= 84.2%
Waist circumference (cm)	24 (<1%)	Mean: 62.5<br>Range: 32.1-121.5	mean: 62.5<br>range: 32.1-121.5
Height (m)	24 (<1%)	mean: 1.41<br>range: 1.10-1.74	mean: 1.41<br>range: 1.10-1.74
Adolescents			
Parental education	563 (12%)	>compulsory education[a]= 76.2%	>compulsory education[a]= 77.5%
Waist circumference	411 (9%)	mean: 73.2<br>range: 38.0-125.9	mean: 73.5<br>range: 38.0-125.9
Height	21 (<1%)	mean: 1.67<br>range: 1.28-1.98	mean: 1.67<br>range: 1.28-1.98

[a] Percent (%) of which one or both parents have completed any post-compulsory education.

Table S3: Association (unstandardized regression coefficients and 95%CI) between birth weight and cardiometabolic risk factors, and interaction with MVPA (p-value), complete case analyses

	MODEL 1[a]		MODEL 2[b]		
		Association		Association	Birth weight x MVPA
	n	B (95% CI)	n	B (95% CI)	p-value
Children					
SBP (mmHg)	4020	-1.15(-1.55, -0.74)	4015	-1.35 (-1.72, -0.98)	0.499
DBP (mmHg)	4019	-0.69 (-0.93, -0.46)	4014	-0.77 (-1.03, -0.51)	0.130
LDL– cholesterol (mmol/l)	3163	0.03(-0.00, 0.07)	3143	0.01 (-0.01, 0.04)	0.975
HDL- cholesterol (mmol/l)	3167	-0.02 (-0.05, 0.01)	3147	0.00 (-0.03, 0.03)	0.967
Triglycerides (mmol/l)	3148	-0.01 (-0.03, 0.01)	3128	-0.03(-0.05, -0.02)	0.786
HOMA-IR (score)	3052	-0.01 (-0.05, 0.03)	3032	-0.07 (-0.11, -0.03)	0.789
Waist circumference (cm)	4449	1.90 (1.57, 2.23)		-	0.003
Clustered risk score[c]	3022	-0.01 (-0.06, 0.04)	3010	-0.08 (-0.13, -0.04)	0.696
Adolescents					
SBP (mmHg)	3919	-1.66 (-2.45, -0.87)	3625	-1.97 (-2.82, -1.13)	0.747
DBP (mmHg)	3919	-0.41 (-0.74, -0.08)	3625	-0.45 (-0.78, -0.12)	0.481
LDL cholesterol (mmol/l)	2507	-0.00 (-0.05, 0.05)	2320	-0.01 (-0.05, 0.04)	0.332
HDL- cholesterol (mmol/l)	2507	-0.02 (-0.04, -0.01)	2320	-0.01 (-0.03, 0.00)	0.147
Triglycerides (mmol/l)	2506	0.00 (-0.01, 0.01)	2319	-0.02(-0.03, -0.01)	0.789
HOMA-IR (score)	2500	0.01 (-0.05, 0.07)	2313	-0.04 (-0.10, 0.01)	0.718
Waist circumference (cm)	3673	1.73 (1.15, 2.30)		-	0.954
Clustered risk score[c]	2486	0.00 (-0.04, 0.04)	2302	-0.05 (-0.08, -0.01)	0.899

DBP- Diastolic blood pressure; HDL- High density lipoprotein; HOMA-IR- Homeostasis Assessment Model (HOMA2); LDL- Low density lipoprotein; MVPA –Moderate to vigorous physical activity; SBP- Systolic blood pressure

[a] Model 1: Adjusted for highest parental education, sex and age. SBP and DBP adjusted for height instead of age.

[b]Model 2: Adjusted for model 1 and waist circumference

[c]Clustered cardiometabolic risk score calculated from summing standardized values for MAP (mean arterial blood pressure), triglycerides, LDL/HDL-ratio and fasting insulin, divided by 4 (number of variables)
